# Regulation of Inflammatory Chemokine Receptors on Blood T Cells Associated to the Circulating Versus Liver Chemokines in Dengue Fever

**DOI:** 10.1371/journal.pone.0038527

**Published:** 2012-07-16

**Authors:** Luzia Maria de-Oliveira-Pinto, Cíntia Ferreira Marinho, Tiago Fajardo Povoa, Elzinandes Leal de Azeredo, Luiza Assed de Souza, Luiza Damian Ribeiro Barbosa, Ana Rita C. Motta-Castro, Ada M. B. Alves, Carlos André Lins Ávila, Luiz José de Souza, Rivaldo Venâncio da Cunha, Paulo Vieira Damasco, Marciano Viana Paes, Claire Fernandes Kubelka

**Affiliations:** 1 Laboratório de Imunologia Viral, IOC, FIOCRUZ, Rio de Janeiro, Brazil; 2 Laboratório de Biotecnologia e Fisiologia de Infecções Virais, IOC, FIOCRUZ, Rio de Janeiro, Brazil; 3 Centro Regional de Referência em Dengue, Campos dos Goytacazes, Rio de Janeiro, Brazil; 4 Setor Hemonúcleo, Universidade Federal do Mato Grosso do Sul, Mato Grosso do Sul, Brazil; 5 Hospital Universitário Pedro Ernesto, Universidade Estadual do Rio de Janeiro, Rio de Janeiro, Brazil; University of Leuven, Rega Institute, Belgium

## Abstract

Little is known about the role of chemokines/chemokines receptors on T cells in natural DENV infection. Patients from DENV-2 and -3- outbreaks were studied prospectively during the acute or convalescent phases. Expression of chemokine receptor and activation markers on lymphocyte subpopulations were determined by flow cytometry analysis, plasma chemokine ligands concentrations were measured by ELISA and quantification of CCL5/RANTES^+^ cells in liver tissues from fatal dengue cases was performed by immunochemistry. In the acute DENV-infection, T-helper/T-cytotoxic type-1 cell (Th1/Tc1)-related CCR5 is significantly higher expressed on both CD4 and CD8 T cells. The Th1-related CXCR3 is up-regulated among CD4 T cells and Tc2-related CCR4 is up-regulated among CD8 T cells. In the convalescent phase, all chemokine receptor or chemokine ligand expression tends to reestablish control healthy levels. Increased CCL2/MCP-1 and CCL4/MIP-1β but decreased CCL5/RANTES levels were observed in DENV-patients during acute infection. Moreover, we showed an increased CD107a expression on CCR5 or CXCR3-expressing T cells and higher expression of CD29, CD44^HIGH^ and CD127^LOW^ markers on CCR4-expressing CD8 T cells in DENV-patients when compared to controls. Finally, liver from dengue fatal patients showed increased number of cells expressing CCL5/RANTES in three out of four cases compared to three death from a non-dengue patient. In conclusion, both Th1-related CCR5 and CXCR3 among CD4 T cells have a potential ability to exert cytotoxicity function. Moreover, Tc1-related CCR5 and Tc2-related CCR4 among CD8 T cells have a potential ability to exert effector function and migration based on cell markers evaluated. The CCR5 expression would be promoting an enhanced T cell recruitment into liver, a hypothesis that is corroborated by the CCL5/RANTES increase detected in hepatic tissue from dengue fatal cases. The balance between protective and pathogenic immune response mediated by chemokines during dengue fever will be discussed.

## Introduction

Dengue fever (DF) is usually a self-limiting yet debilitating febrile illness, but occasionally it may present severe clinical manifestations that are life-threatening and are characterized by increased vascular permeability, thrombocytopenia, hemorrhages and shock [1]. Infection with one of the four Dengue virus (DENV) serotypes presumably leads to a long lasting protective immunity against the corresponding serotype but not against others. In fact, severe DF is most often observed in individuals experiencing a secondary infection with a heterologous serotype [2], and it has been postulated that serotype cross-reactive antibodies and memory T cells are involved in the pathogenesis [3], [4]. Serotype-cross-reactive T cells are preferentially activated during a second DENV infection in a phenomenon termed as “original antigenic sin”, indicating a pathogenic role of T cells during sequential DENV infections [5]. These cross-reactive T cells have exhibit suboptimal degranulation and altered cytokine production [6], [7], [8]. In fact, an aberrant cytokine production by T cells could contribute to worsen disease, as high levels of certain proinflammatory mediators are suspected to cause endothelial cell activation or damage, leading to plasma leakage, a hallmark of severe DF and shock [9]. However, another study found the breadth and magnitude of the T cell response during secondary DENV infection were not significantly associated with disease severity [10]. Consequently, the role of T cells in protection versus pathogenesis during DENV infections still presents some unclear aspects.

Inflammatory chemokine receptors such as CCR5, CXCR3, and CCR4 are expressed in inflamed tissues by resident and infiltrating cells upon stimulation by pro-inflammatory cytokines or during contact with pathogenic agents. Moreover, such chemokines are secreted early after infection in response to the activation of pattern recognition receptors on epithelial, stromal and immune cells. They recruit the initial wave of innate immune effector cells, including neutrophils, monocytes, natural killer (NK) cells, and NKT cells, all expressing inflammatory chemokine receptors and immature dendritic cells (DC) providing the link between innate and adaptive immunity. After antigen-specific activation of lymphocytes by activated DC inflammatory chemokines attract then antigen-specific effector T cells to the inflammatory site [11]. At the same time regulatory cells are also recruited and the balance between effector and regulatory cell recruitment determines the outcome of the local inflammation. Chemokines and their receptors also undergo post-translational modifications which alter their functions allowing them to provide almost unlimited potential receptor ligand pairs to bring exquisite specificity to the control of leukocyte homing and positioning in tissues [12]. The deregulated expression of chemokines and their receptors is involved in the development of many human diseases, including autoimmune and chronic inflammatory diseases as well as immunodeficiency and cancer [13]. Specific chemokine receptors, expressed on activated lymphocytes, are also known to be associated with T-helper (Th) phenotypes. Summarizing, CD4 T cells can be divided into functionally polarized subsets based on the cytokines they produce: Th1 cells produce mainly type 1 cytokines, including interferon-gamma (IFN-γ) and interleukin-2 (IL-2), and promote cell-mediated immune responses, whereas Th2 cells secrete type 2 cytokines, including IL-4, IL-5, IL-6, IL-10, and IL-13, and enhance humoral immune responses [14], [15], [16]. Moreover, a similar cytokine secretion pattern has been observed for CD8 cytotoxic T cells that are designated Tc1 and Tc2 cells [14], [15], [17], [18]. In particular, CC chemokine receptor (CCR) 5 and CXC chemokine receptor (CXCR) 3 are usually associated with a Th1 phenotype, while Th2-associated chemokine receptor CCR2 and CCR4 have been reported [19]. Moreover, chemokine receptors are also useful to discriminate naïve, memory and effector subsets in the human CD4 and CD8 T cells. For example, CCR5 is predominantly expressed on memory CD8 T cells, and its expression decreases during differentiation from memory to effector T cells [20], while CXCR3 on CD4 and CD8 T cells are strongly enhanced when these cells are activated [21], [22], [23]. In addition, CCR4 is selectively expressed on the majority of peripheral memory Th2 cells [24], [25] and a report characterizing specifically CCR4^+^CD8^+^ T cells, described that these cells have the ability to produce multiple cytokines including IL-4.

The regulation of chemokine receptor expression and chemokine production by lymphoid cells in response to DENV has been studied. Increased level CCL2/MCP-1, which attracts monocytes and is strongly involved in the reduction of the tight junctions of vascular endothelial cells, was found in patients suffering from severe DF [26]. In vitro infection of immature dendritic cells (imDC) with DENV-2 led to induction of CCL11/Eotaxin, CCL2/MCP-1, CCL5/RANTES, CCL3/MIP-1α and CXCL10/IP-10 [27], [28]. Several chemokines are produced by the endothelial cells infected in vitro by DENV, such as CCL2/MCP-1, CCL5/RANTES and CXCL8/IL-8 [29], [30], [31]. It was recently demonstrated an increased CXCR3 expression on T cells during DENV acute infection, but not in the convalescent phase [32]. In this same study, the presence of activated T cells co-cultured with DENV-infected DC has led to an increased production of IFN-induced chemokines such as CXCL9/MIG, CXCL10/IP-10, and CXCL11/I-TAC, which are CXCR3 ligands and were also observed later in the plasma from patients affected by severe DF. This hypothesis was confirmed in the work of Chau and collaborators that showed that those patients with shock showed higher plasma levels of the same chemokines than those severe patients who had not shock except for CCL5/RANTES [33].

T cells are recruited to inflammation sites by the coordinated expression of several homing molecules, including integrins [34], [35]. Integrins are a family of membrane glycoproteins that mediate cell adhesion to extracellular matrix components and to other cells [36], [37] and are composed of two noncovalently associated type I transmembrane glycoproteins [38]. Among them are CD29 (β_1_) integrin that include VLA-4 (CD49d/CD29) [13], [39] Similarly, T cell migration CD44 receptor is a glycoprotein ascribed for having a role in lymphocyte homing to sites of inflammation [40], [41] Furthermore, signals emanating from the cytokine receptor for IL-7 (CD127) are particularly critical for the generation and long-term maintenance of memory CD8 T cells [42] and are of interest in understanding T lymphocyte functions during infections.

Little is known about chemokine receptors on T cells during natural DENV infection and what would their role be in the disease severity. Thus, this paper aims to evaluate the profile of CCR5-, CXCR3- and CCR4-expression on CD4 and CD8 circulating T cells and their cognate ligands in blood from adults with dengue fever in Brazil. Moreover, we specifically analyzed CCL5/RANTES expression in liver from patients who died during DENV infection.

## Materials and Methods

### Study Population

We prospectively included patients infected with DENV in the acute (2–9 days after first symptoms) or convalescent phases (up to 15 days) from 2007 to 2008 DENV-2 and -3 outbreaks. Patients were assisted at Hospital Universitário Pedro Ernesto at Universidade Estadual do RJ, Centro de Referência em Dengue at Campos dos Goytacazes and Universidade Federal do Mato Grosso do Sul. Patients had DENV infection confirmed by anti-DENV enzyme-linked immunosorbent assay (ELISA)-IgM (Panbio, Inc., USA). Demographical, clinical and laboratorial data from the DF without Warning Signs (DF), DF with Warning Signs plus Severe (WS/Severe) and convalescent patients enrolled in this study are shown in Table 1. Patients typically developed high-grade fever suddenly. This acute febrile phase usually lasts 2–7 days and is often accompanied by facial flushing, skin erythema, generalized body ache, myalgia, arthralgia and headache. Anorexia, nausea and vomiting were common. These clinical features are characteristic as non-severe dengue cases. Monitoring for warning signs and other clinical parameters are crucial for recognizing progression to the critical phase and it can clinically distinguish dengue from non-severe and severe dengue cases. The warning signs were assessed as clinical parameters such as abdominal pain or tenderness, persistent vomiting, clinical fluid accumulation, mucosal bleed, lethargy, restlessness, liver enlargement >2 cm associated to laboratory parameters as increase in haematocrit (HCT) concurrent with rapid decrease in platelet count. Mild hemorrhagic manifestations like petechiae and mucosal membrane bleeding (e.g. nose and gums) may be observed. Patients require emergency treatment and urgent referral when they are in the critical phase of disease, i.e. when they have severe plasma leakage leading to dengue shock and/or fluid accumulation with respiratory distress, severe hemorrhages showed by massive vaginal bleeding (in women of childbearing age) or gastrointestinal bleeding and/or severe organ impairment (hepatic damage, renal impairment, cardiomyopathy, encephalopathy or encephalitis) [43]. All patients recovered from the illness uneventfully and survived to hospital discharge.

**Table 1:** Demographic and clinical characteristics of DENV confirmed patients.

		Classification		
Characteristic	Controls^n = 16^	DF^n = 33^	WS/Severe^n = 40^	Convalescent^n = 26^
**Demographic data**				
Age in years(mean ± SD)	31.9±11	37.3±15.1	38.2±15.7	42.8±20.4
Gender (F:M)	14∶2	15∶18	20∶20	7∶19
Illness Day^a^ (mean ± SD)	ND	4.6±2.5	3.4±2.1	>15–60
Primary history (%)	ND	81	78.1	ND
Secondary history (%)	ND	19	21.9	ND
**Clinical sign and symptoms**				
Effusion^c^ (%)	0	0	20	ND
Hypotension^d^ (%)	0	4.3	56.4	ND
**Laboratory parameters**				
Platelets × 10^3^/mm^3^ (mean ± SD)	ND	137.4±57.9	92.8±84.8*	160.1±90.4^&^
Haematocrit % (mean ± SD)	ND	41.6±4.2	41.9±6.5	44.1±2.8^&^
Total Leukocyte count/mm^3^ (mean ± SD)	ND	3733.3±1639.4	3532±1428	5350±1152.6^&^
Total Lymphocyte count/mm^3^ (mean ± SD)	ND	1172.7±637.7	877.8±270.6	1809.3±463.5^&^
CD4+CD8- T cells % (mean ± SD)	29.9±4.8	23.8±10.9	25.8±11.8	32±10.8
CD8+CD4- T cells % (mean ± SD)	20.9±4.1	25±13.1	21±8.1	18.5±6.8
AST (IU/L) (mean ± SD)	ND	105.1±117.6	135.9±108	139.8±204
ALT (IU/L) (mean ± SD)	ND	114.9±116.4	110±95.9	121±131

DF, DF Without WS; WS/Severe, DF With WS+ Severe; ND, Not determined.

Illness Day^a^ corresponds to the days of the start of any symptoms until the moment when the patient was interviewed; Bleeding^b^ includes skin haemorrhages, epistaxis, gingival, gastrointestinal, urinary tract haemorrhage or metrorrhagia; Fluid leakage^c^ signs of plasma leakage (pleural or pericardial effusion, ascites); Hypotension^d^ defined by pulse pressure below 20 mm Hg and/or hypotensive for age; Convalescent patients who have early symptoms for more than 15 days. Include some of the patients in the acute phase. Statistical differences were assessed by the Mann Whitney U test to evaluate differences in different parameters between controls and DENV-patients.

* represents P values <0.05 between DF versus WS/Severe and & represents P values <0.05 between Acute versus Convalescent patients.

### Human Fatal Cases

The human hepatic tissues analyzed were obtained from four fatal dengue cases, during the outbreak of DENV-3 in 2002 in Rio de Janeiro. All patients, ranging from 21 to 63 years old, presented fever, myalgia and hemorrhagic manifestations. The dengue diagnosis was confirmed by positive serum IgM antibodies. The negative control was obtained from a female patient, of 58 years old who died of stroke, a female of 23 years old, who did not die of dengue and man of 70 years of age who died of trauma and septic shock.

### Ethical Procedures

All procedures performed during this work were approved by the Ethics Committee of the Oswaldo Cruz Foundation nr. 0111/00 for non-fatal dengue cases and nr. 434/07 for fatal dengue cases, Brazilian Health Ministry (recognized by the Brazilian National Ethics Committee). Signed informed consent was obtained from all patients. The control group consisted of healthy subjects with negative anti-IgM DENV.

### Blood Mononuclear Cell Preparation

About twenty ml of peripheral venous blood were taken into Na-heparin anti-coagulated tubes from patients and controls. PBMC were separated from blood samples by performing Ficoll-Hypaque (d = 1077 g/ml; Sigma) and centrifuged at 400 g for 30 min. The PBMC layer was washed twice in RPMI 1640 medium. The viability of PBMC was greater than 95% after Trypan blue exclusion. Approximately 10^6^ PBMCs were re-suspended in 1 ml of solution destined for freezing (80% inactivated FBS (Gibco, Invitrogen Co.) plus 20% DMSO (Sigma)) and stored initially at −70°C for 24 h before introduction into liquid nitrogen, and aliquots were cryopreserved for later study.

### Reagents and Monoclonal Antibodies

Frequency of lymphocyte subpopulations (CD4+ and CD8+ T cells) were enumerated and for chemokine receptor or activation markers expression, five or six-color flow cytometry method was performed using the following monoclonal antibodies: anti-CD4- Allophycocyanin conjugated cyanine dye (APC Cy7) (BioLegend), anti-CD8- phycoerythrin conjugated a cyanine dye (MW 1.5 kDa) (PE Cy5) (BD Biosciences), anti-CCR5- phycoerythrin (PE), anti-CXCR3- peridinin chlorophyll (PerCP) (all R&D Systems, Abingdon, UK) and anti-CCR4-APC (e-BioSciences). The activation markers used were fluorescein isothiocyanate (FITC)-conjugated with: anti-CD29, anti-CD44 (both Dako), and anti-CD38 (Caltag). The expression of the CD107a marker was performed with anti-CD107 to Alexa Fluor® 647 Conjugate from R&D Systems. Appropriate matched isotype control antibodies were used to discriminate between positive and negative populations/subsets.

### Flow Cytometric Determination on Peripheral Blood T Lymphocytes

Chemokine receptor and activation markers expression on lymphocyte subpopulations/subsets was determined by staining 100,000 cells of peripheral blood with appropriate combinations of monoclonal antibodies to compare chemokine receptors and activation markers expression on CD4+ (helper) and CD8+ (cytotoxic) T lymphocytes. PBMCs were initially suspended in PBS. Then, cells were incubated on ice for 40 min in the dark with flourochrome-conjugated monoclonal antibodies, washed twice with PBS, and fixed in 1% paraformaldehyde. Fixed cells were kept in the dark until acquisition. A minimum of 10,000 T cells gated events were acquired using a CyAn flow cytometer (Dako) and analysis was performed using Flow Jo v.7.6.1 software.

### Plasma Chemokine Determinations

Plasma chemokine concentrations of the main CCR5 ligands (CCL4/MIP-1beta and CCL5/RANTES) and CCR4 ligand (CCL2/MCP-1) were measured in the variable timing cohort by enzyme-linked immunosorbent assays (ELISA) (Quantikine, R&D Systems).

### Immunohistochemistry Procedure

Liver tissues from the human necropsies were used for immunochemistry assays. Formalin-fixed and paraffin-embedded tissues were cut (4 µm), deparaffinized in xylene and rehydrated with alcohol. Antigen retrieval was performed by heating the tissue in a citrate buffer. Such tissues were then blocked for endogenous peroxidase with hydrogen peroxidase in methanol and rinsed in Tris-HCl (pH 7.4). To reduce non-specific binding, sections were incubated with bovine fetal serum for 30 min. Samples were then incubated overnight with human CCL5/RANTES-specific IgG polyclonal antibodies (Santa Cruz Biotechnology), followed by treatment with secondary polyvalent horseradish peroxidase-conjugated antibody (Spring Bioscience). For negative control, samples were incubated with the secondary horseradish peroxidase-conjugated antibody only. Reaction was revealed with diaminobenzidine as chromogen and the sections were counterstained in Meyer’s hematoxylin. Slides were evaluated using a Nikon ECLIPSE E600 microscope with the Cool SNAP-Procf Color camera coupled. In each case, 50 images were randomly acquired in 400×magnification by Image Pro software, and the percentage of positive RANTES cells was quantified (number of positive RANTES cells/50 fields).

### Statistical Analysis

Statistical analysis was performed using GraphPad prism software, version 4.0 (GraphPad Software Inc., San Diego, CA, USA). The Mann–Whitney U-test was used to compare non-parametric data from patients and controls. Correlation was estimated by Spearman regression analysis. A P value of <0·05 was taken as being significant for all statistical analyses.

## Results

### DENV-infected Patients and Controls

Demographical, clinical and laboratorial data from the DF, WS/Severe and convalescent patients enrolled in this study are shown in Table 1. Age, duration of disease, hematocrit, serum alanine or aspartate aminotransferase (ALT or AST) levels at admission were comparable among patients in the DF, WS/Severe and convalescent group. It is interesting to note that while the CD4 or CD8 T cell frequencies were not different among these three groups, the lymphocyte counts were significantly lower during DF as compared to convalescent group as observed as well for the total leukocytes. Moreover, during convalescence period, patients rapidly recover and present significantly higher leukocyte counts than acute DENV-patients (convalescent vs. DF, p = 0.0476 and WS/Severe, p = 0.0426). Similar effect is also observed for platelet counts. The WS/Severe patients had shown even lower platelet counts than those of DF patients (p = 0.0187); platelet counts were lower at acute phase than during convalescence.

To gain further insight into changes occurring during dengue fever, various laboratory determinations routinely taken that could theoretically affect disease severity were analyzed with respect to their associations. Spearman analysis performed showed that the illness day correlated positively with platelet counts (r = 0.3982, p<0.001, n = 66) and inversely with CD8^+^ frequencies (r = −0.3173, p<0.04, n = 44). These findings indicate that as disease progresses, circulating platelets and leukocytes that are initially diminished tend to increase towards normal levels while the CD8^+^ T cells that were transitorily elevated started to decrease during the course of disease. In addition, lower platelet counts were correlated with higher serum AST levels (r = −0.5960, p<0.0001, n = 40). No correlation was found between the day of the illness or platelet counts with neither hematocrit, AST levels nor CD8^+^ cell frequencies.

### CCR5 Expression is Increased on Both CD4 and CD8 T Cells, While CXCR3 and CCR4 are Differentially Up-regulated Respectively among CD4 and CD8 T Cells in DENV-infection

**Figure 1:**
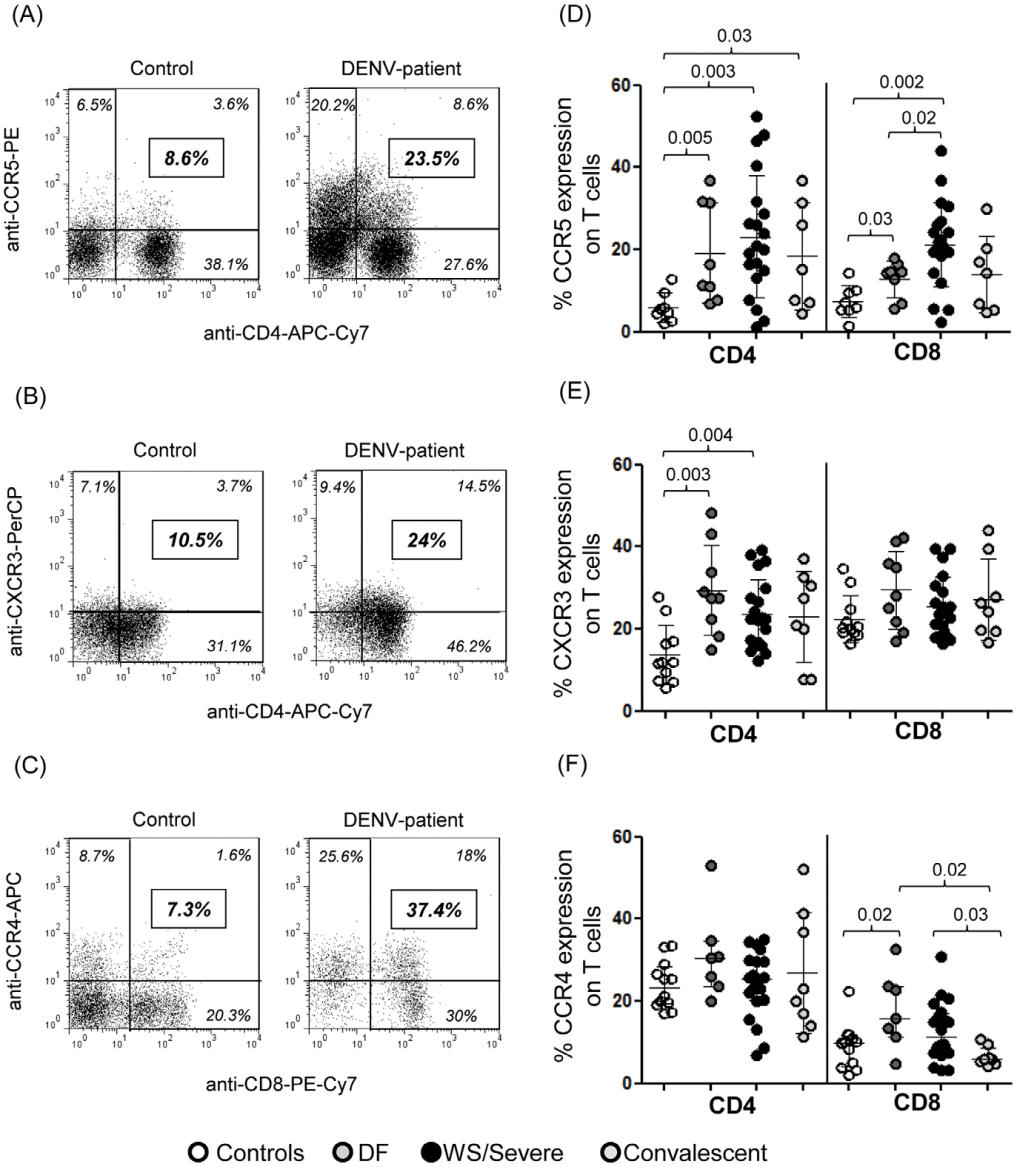
Expression of CCR5, CXCR3 and CCR4 on T cells from DENV-patients and healthy controls. Representative dot plots showing the expression of CCR5 (A), CXCR3 (B) and CCR4 (C) on CD4 or CD8 T cells in one healthy control and one DENV-patient. The values in italics in each region of the quadrants indicate the values of cells’ frequency of the quadrants. The bold-, italic and framed numbers on the right indicate the percentages of chemokine receptors among T cells. (D) Percentage of CCR5-expressing among CD4+ or CD8+ T cells; (E) percentage of CXCR3-expressing among CD4+ or CD8+ T cells; (F) percentage of CCR4-expressing among CD4+ or CD8+ T cells. Horizontal bars indicate the mean values, standard deviation for each population. Appropriate matched isotype control antibodies were used to discriminate between positive and negative populations. The Mann–Whitney U-test was used to analyze differences between control and patient groups. Statistically significant P values for differences between patients and controls are shown above the figures.

Initially, representative dot plots in Figure 1 show the increased expression of CCR5 on CD4 (Fig. 1A), CXCR3 on CD4 (Fig. 1B) and CCR4 on CD8 (Fig. 1C) T cells in one DENV-patient compared to one healthy control. Our data indicate that CCR5+ cell rates present among all CD4 T cells as well as among all CD8 T cells were markedly higher during dengue disease as compared to controls. An increasing trend was observed across the three groups namely, from controls to DF, WS/Severe while a decreasing trend was observed in the convalescent group, but still significantly elevated on CCR5+ among CD4 T cells. Interestingly, CCR5+ among CD8 T cells was significantly higher in the WS/Severe as compared with DF (Fig. 1D) during acute phase. The CXCR3+ frequency among CD4 T cells was significantly higher in all acute patients compared to controls, irrespective of severity whereas the CXCR3+ percentage among CD8 T cells was similar in all DF groups and controls (Fig. 1E). Again, the CCR4+ percentage among CD4 T cells was similar in studied groups. Differently CCR4+ among CD8 T cells was significantly higher in DF compared to controls and convalescent groups and WS/Severe showed increased frequency of CCR4+ among CD8 T cells compared to convalescent group (Fig. 1F).

### Differential Circulating Chemokine Levels from DENV-infected Patients

**Figure 2:**
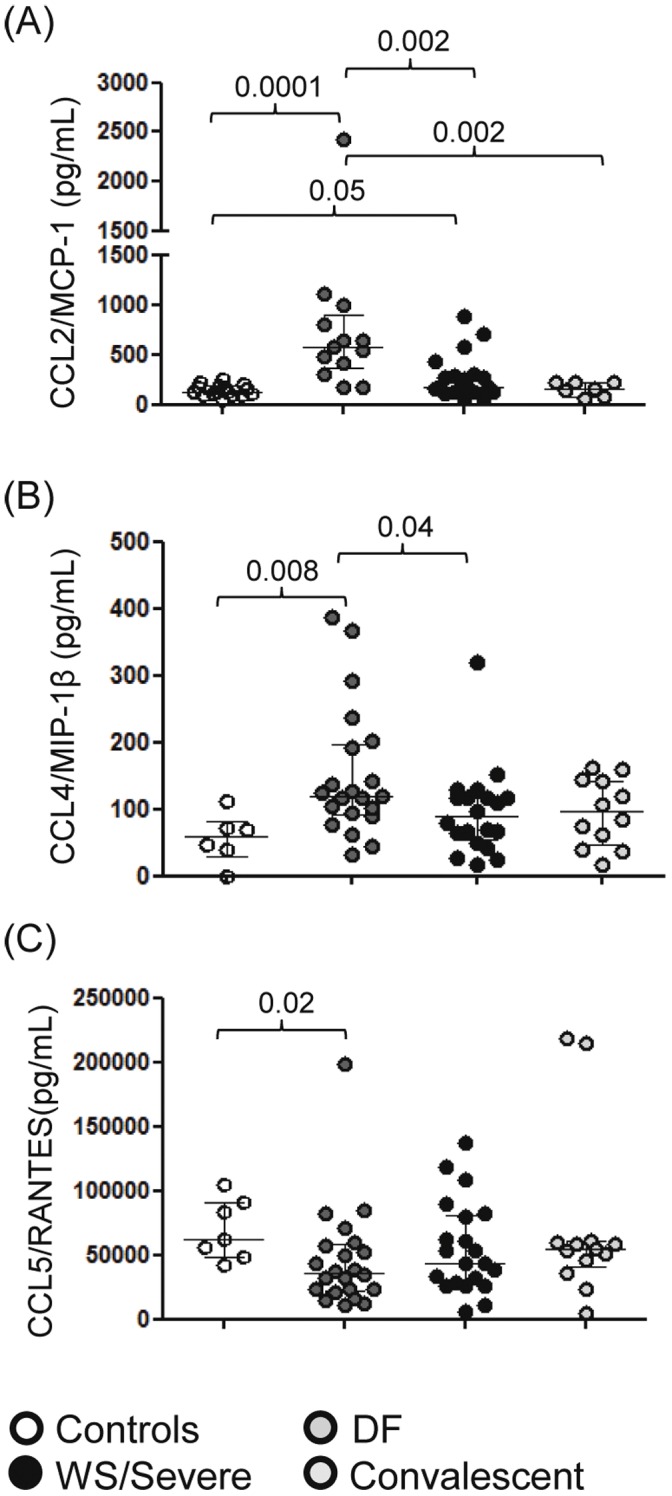
Circulating CCL2/MCP-1, CCL4/MIP-1β and CCL5/RANTES are differentially expressed DENV-patients. Each individual is represented, comparing healthy controls, acute or convalescent DENV-patients. The Mann–Whitney U-test was used to analyze differences between control and patient groups. Statistically significant P values for differences between patients and controls are given above the figures.

We analyzed CCR5 ligand CCL2/MCP-1 levels in plasma from DENV-patients. As shown in Fig. 2A, patients with acute DF showed higher plasma CCL2/MCP-1 levels than normal controls and convalescent patients. In addition, plasma CCL2/MCP-1 levels in patients with WS/Severe were statistically lower when compared to those patients DF. Convalescent patients had their CCL2/MCP-1 plasma concentration back to normal since they did not differ significantly from those found in controls. Another CCR5 ligand, the CCL4/MIP-1β was present in significantly higher circulating levels in DF patients than in controls but also higher than levels in WS/Severe (Fig. 2B). Moreover, CCR5 plasma ligand CCL5/RANTES levels were not statistically different among the three groups: DF, WS/Severe and convalescent despite these levels were lower than controls with statistical significance in the DF (Fig. 2C).

### CD8 T Lymphocytes Expressed Both CD107a and CD38 Cytotoxicity/activation Markers, While CD4 Expressed Only CD107a

**Figure 3:**
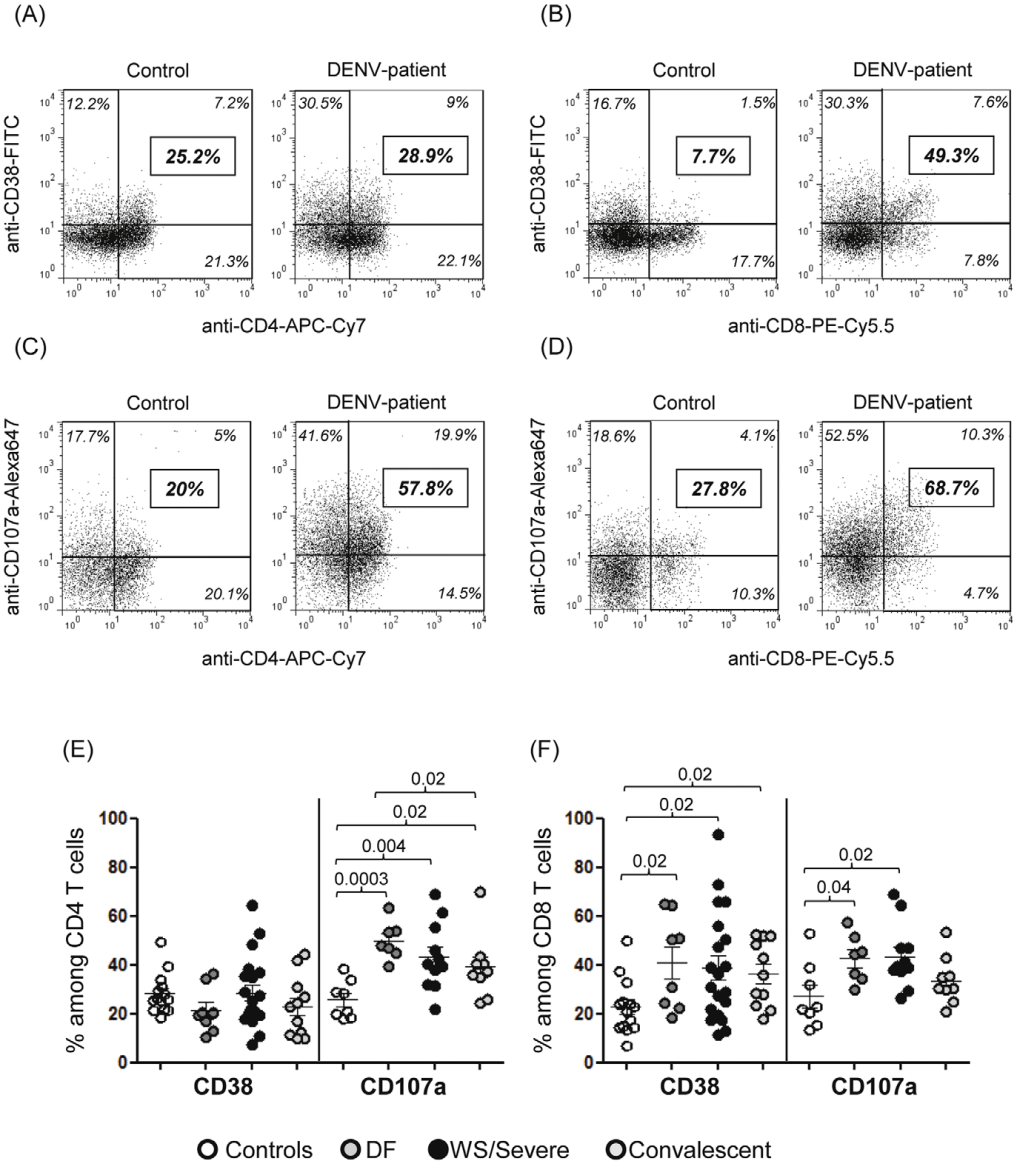
Phenotype of CD4 and CD8 T cells from DENV-patients. Representative flow cytometry dot plots comparing the frequency of CD38+ among CD4 and CD8 T cells (A–B) and CD107a+ among CD4 and CD8 T cells (C–D) and in one healthy control and one DENV-patient. The values in italics in each region of the quadrants indicate the values of the cells’ frequency of the quadrants. The bold-, italic and framed numbers on the right indicate the percentages of marker among T cells. Appropriate matched isotype control antibodies were used to discriminate between positive and negative populations. (E) Percentage of CD38 or CD107a-expressing among CD4+; (F) Percentage of CD38 or CD107a-expressing among CD8+ T cells. Horizontal bars indicate the mean values, standard deviation for each population. The Mann–Whitney U-test was used to analyze differences between control and patient groups. Statistically significant P values for differences between patients and controls are shown above the figures.

The proportion of CD4 T cells expressing CD38 was not altered in dengue cases compared to controls. Interestingly, there was a significant increased proportion of CD4 T cells expressing CD107a in all dengue status and still in convalescent group as compared to healthy individuals (Fig. 3E). In contrast, dengue cases showed significantly higher CD8 T cells expressing CD38 in all disease status and still in convalescent group compared to controls. Similarly, dengue cases showed significantly higher CD8 T cells expressing CD107a in acute DENV-infected patients but not in convalescent group when compared to control individuals (Fig. 3F). Representative flow cytometer analysis showed this non-altered CD38+ cell expression among CD4 but increased CD38+ among CD8 T cells (Fig. 3A–B), while an increased CD107a+ expression was observed in both T cells (Fig3. C–D) from one healthy control and one DENV-patient.

### Increased CD107a Expression on CCR5 or CXCR3-expressing T Cells in Dengue Disease Compared to Controls

**Figure 4:**
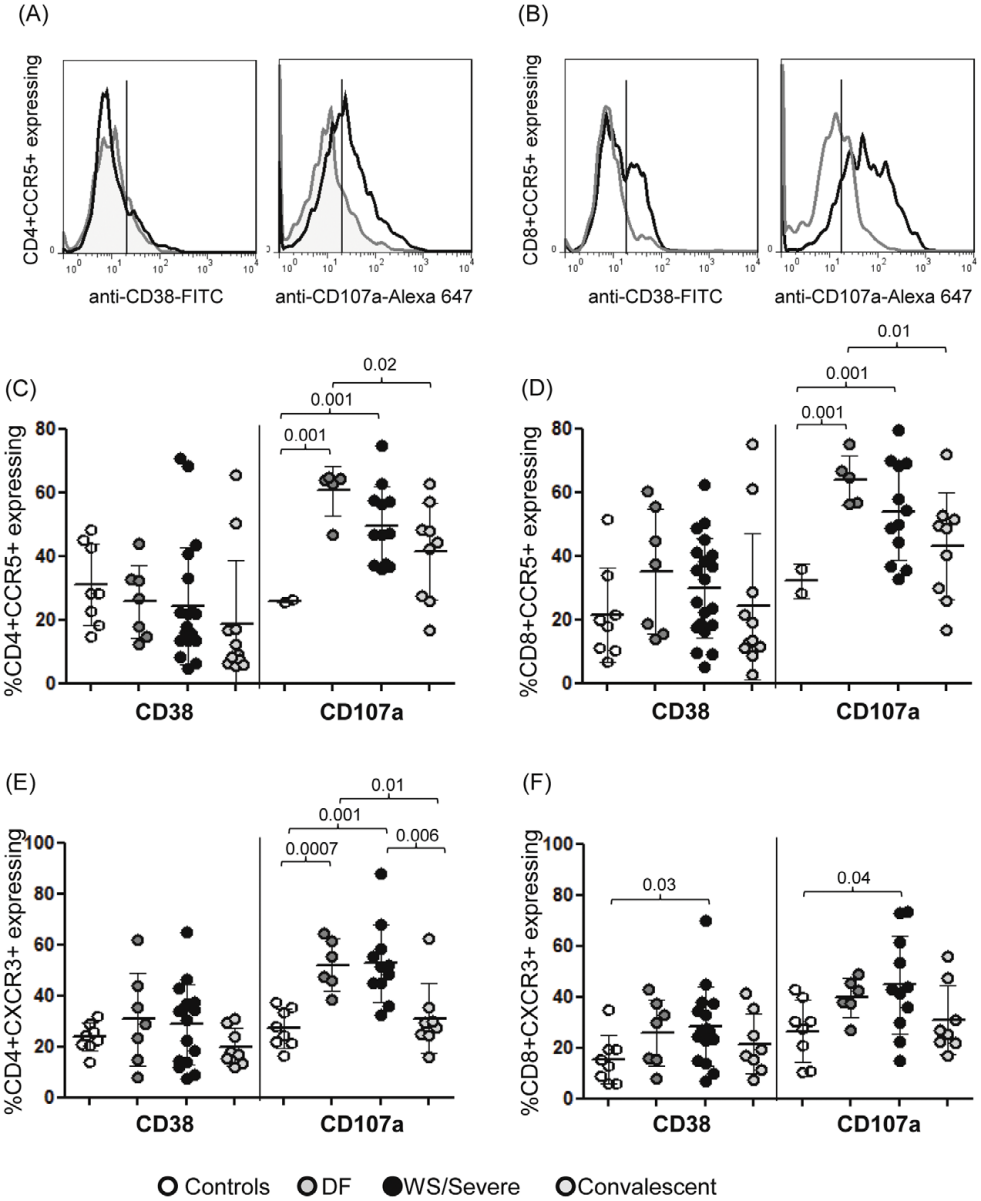
Phenotype of CCR5- or CXCR3 among CD4 and CD8 T cells from DENV-patients. Representative histogram plots comparing (A) the frequency of CD38+ or CD107a+ among CD4+CCR5+ T cells and (B) the frequency of CD38+ or CD107a+ among CD8+CCR5+ T cells between one healthy control (gray line) and one DENV-patient (black line). Vertical bars indicate the positive values based an appropriate matched isotype control antibodies. Percentage of CCR5+CD4+ (C) or CCR5+CD8+ T cells (D) expressing among CD38+ or CD107a+; percentage of CXCR3+CD4+ (E) or CXCR3+CD8+-T cells expressing among CD38+ or CD107a+. Horizontal bars indicate the mean values, standard deviation for each population. The Mann–Whitney U-test was used to analyze differences between control and patient groups. Statistically significant P values for differences between patients and controls are shown above the figures.

Regarding the expression of CD38 on CCR5+ on both T cells or CD38 on CXCR3+CD4+ T cells were not altered in acute DENV-patients. However, concerning CD107a expression, it was increased among CCR5+ from both T cells or from CXCR3+CD4+ T cells in acute DENV-patients. The only observed increased frequency of CD38+ or CD107a expression was on CXCR3+CD8+ T cells in WS/Severe group of DENV-patients compared to the control group (Fig. 4A–F). The increase in CD38 expression on CCR5+CD8+ T cells but not in CCR5+CD4+ T cells and yet, an increase in CD107a expression on both T cells expressing CCR5+ were clearly noticed in flow cytometer histogram represented in the upper left panel to the upper right panel in Figure 4 A–B.

### Higher Expression of CD29 or CD44 Activation Markers on T Cells and Lower CD127 Expression on CD8 T Cells

The results obtained demonstrated that in general acute- or convalescent DENV-patients showed ex vivo a high expression of CD29 among CD4 and CD8 T cells, except the WS/Severe group that presented only a slight increase of CD29 among CD8 T cells but not statistically significant when compared to healthy controls. (Table 2) With respect to CD127 expression, it was not altered on CD4 T cells in DENV-patients compared to controls, but a lower expression of CD127 on CD8 T cells was detected mainly in DF and Convalescent group during dengue when compared to healthy controls; in the WS/Severe group this decrease was not significantly noticed. Finally, an increase of CD44 high expression was observed in all groups of DENV-patients, but these increases were biased among CD4 T cells and in DF group on CD8 T cells.

**Table 2:** The detection of CD29, CD127 or CD44^high^ markers on T cells.

	CD4 T cells on			CD8 T cells on		
	CD29	CD127	CD44^high^	CD29	CD127	CD44^high^
Controls	29.3±6.9	66.7±6.6	27.6±13	29.2±8.9	58.7±7.6	17.1±1
DF	53.5±10.7**	70.1±15	45.7±38.9	55.6±22.2*	40.1±7**	44.3±27.7
WS/Severe	42.2±14.6* ^&^	68.5±14.4	53.4±18.4	44.2±18.7	45.3±15.6	45.7±15.8*
Convalescent	43.9±12.5*	71.2±9.7	51.2±14.8	48.9±11.4*	30.1±13.6*	44.1±20.1*

Statistical differences were assessed by the Mann Whitney U test to evaluate differences in parameters between controls and DENV-patients.

* represents P values <0.05 between Controls versus DENV-patients and ^&^between DF vs DF WS/Severe.

### Higher Expression of CD29 or CD44 Markers in CCR4-expressing CD8+ T Cells in Dengue Cases Compared to Controls

Interestingly, in DENV-patients, the subpopulation CCR4+ among CD4 T cells does not appear to modulate the expression of any of the activation markers studied in the course of dengue fever once there was no significant difference compared to healthy controls. Instead, the subset CCR4+CD29+ among CD8 T cells were up regulated during the acute phase, remaining at the stage of convalescence in DENV-patients compared to controls, while an increase of CCR4+CD44^high^ among CD8 T cells was observed in all DENV-patients, but only in WS/Severe patients this increase was statistically significant (Table 3). Representative flow cytometry analysis showed the increased CD29+ and CD44high subpopulation among CCR4+CD8+ T cells in one DENV-patient compared to one healthy control (Table 3).

**Table 3:** The combination of CD29, CD127 or CD44 markers with CCR4-expressing T cells.

	CCR4+ on CD4 T cells			CCR4+ on CD8 T cells		
	CD29	CD127	CD44^high^	CD29	CD127	CD44^high^
Controls	9.1±3.6	16.7±3.6	11.7±7	1.8±0.7	7.8±5.6	2.5±1.7
DF	14.1±6.4	21.4±3.6	17.1±13.3	3.7±1**	8.6±3.1	7.4±4*
WS/Severe	9.9±5.3	19.4±3.2	17.5±5.5	3.6±2.2*	11.2±7.8	9.2±6.1*
Convalescent	12.7±8.2	19.5±5.1	17.7±7.3	2.8±0.9*	5.3±1.4	6.9±3.8

Statistical differences were assessed by the Mann Whitney U test to evaluate differences in parameters between controls and DENV-patients.

* represents P values <0.05 between Controls versus DENV-patients and ^&^between DF vs DF WS/Severe.

### Correlation between Chemokine Receptor Changes or Plasma Chemokine Levels with Frequency of Peripheral T Cells and Platelets

**Figure 5:**
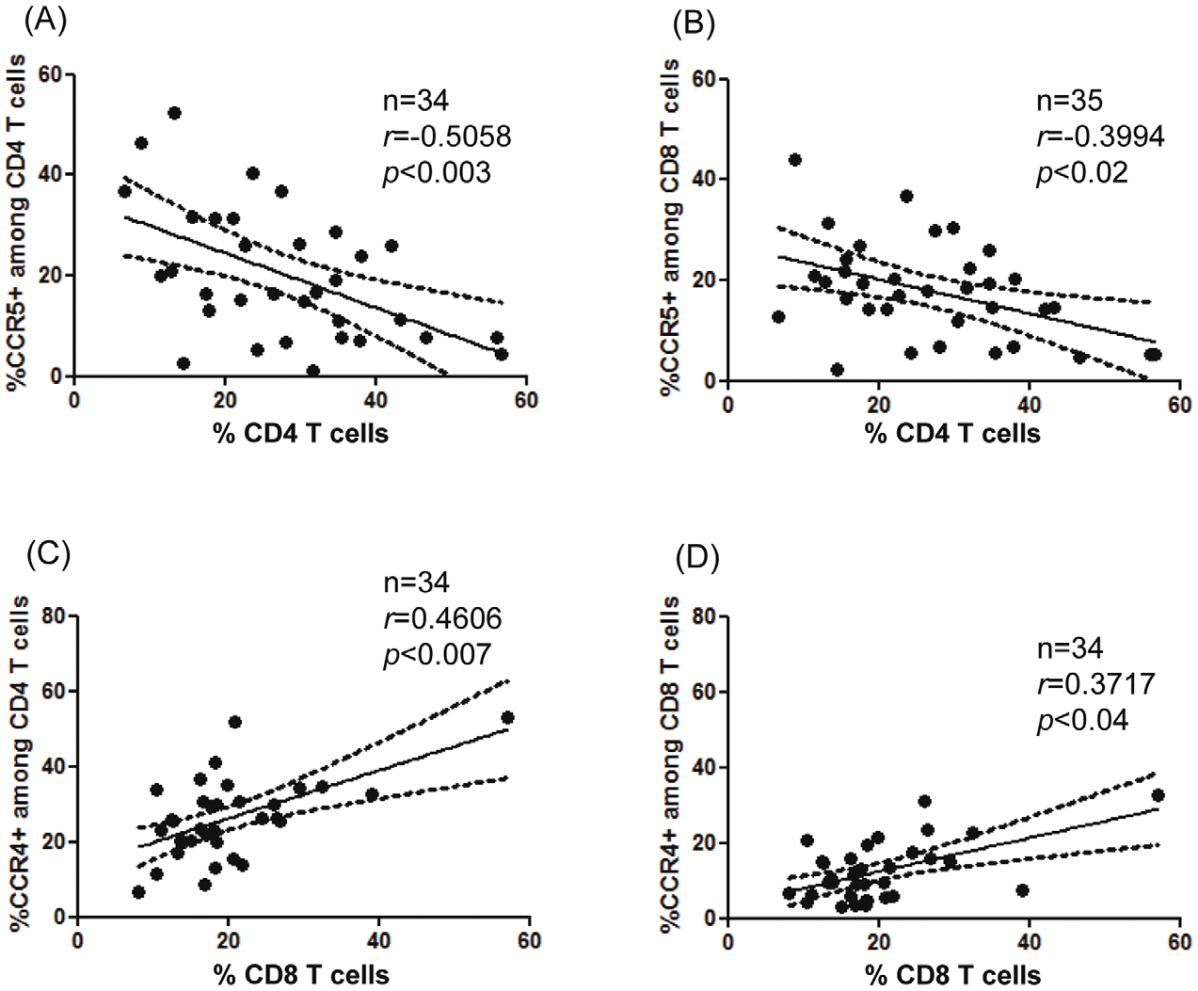
Relationship between chemokine receptor changes with frequency of peripheral T cells. Each acute or convalescent DENV-patients are represented. Correlations were explored using Spearman’s rho test. Statistically significant r values and P are given above the figures.

In the DENV-infected patients, there was an association between the CCR5+ among CD4 or CD8 T cells (Fig. 5A–B) and CCL4/MIP-1β levels with only peripheral CD4 T cells (r = (−) 0.477, p<0.03), but not with CD8 T cells (data not shown). Contrastingly, a positive relationship was found between CCR4+ among CD4 or CD8 T cells only with peripheral CD8 T cells (Fig. 5C–D), not with CD4 T cells (data not shown). No relationship was found between CXCR3+among T cells or CCL2/MCP-1 or CCL5/RANTES chemokines levels measured and CD4+ T cells or CD8+ T cells percentages (data not shown).

The platelet counts correlated positively with the CXCR3+ among CD4 or CD8 T cells (r = 0.393, p<0.05 and r = 489, p<0.02 respectively), CCR4+ among CD4 T cells (r = 0.411, p<0.05) or CCL5/RANTES levels (r = 0.428, p<0.006) (data not shown). No relationship was found between CCR5+ among T cells or CCL2/MCP-1 or CCL4/MIP-1β chemokines levels measured and platelet counts. Also, no relationship was found between any of the other clinical and laboratory parameters (data not shown).

### Quantification of Cells Expressing RANTES in the Liver Tissue of Dengue Fatal Cases

**Figure 6:**
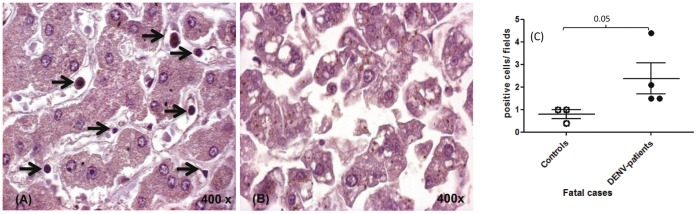
Detection of cells expressing the chemokine CCL5/RANTES in liver tissues from dengue fatal cases. Samples were incubated with CCL5/RANTES-specific antibodies and secondary horseradish peroxidase-conjugated antibodies, revealed with DAB. (A) Liver tissue of one dengue case reacted with CCL5/RANTES-specific antibodies. (B) One dengue case incubated with the secondary antibody only. (C) Quantification of the number of CCL5/RANTES positive cells in the four fatal dengue cases and three fatal non-dengue cases. CCL5/RANTES expressing cells were indicated by →. The Mann–Whitney U-test was used to analyze differences between control and patient groups. Statistically significant P values for differences between patients and controls are shown above the figures.

We detected an increased number of CCL5/RANTES positive cells in the liver tissue collected from three of the four fatal cases mainly in lymphocytes, Kupffer cells and sinusoidal endothelium (Fig. 6A). As expected, we did not detect specific positive staining in a control constituted of a dengue fatal case incubated only with secondary horseradish peroxidase-conjugate antibody (Fig. 6B). The quantification of positive cells showed an increase (ranging from 1.9 to 5.5 fold) in the number of cells expressing CCL5/RANTES on the 50 fields in four dengue fatal cases comparing to data obtained from three non-DENV fatal cases (Fig. 6C).

## Discussion

We investigated here the expression of inflammatory chemokine receptors CCR5, CXCR3, and CCR4 in circulating T cells and the CCR5 ligands during dengue infection, since those receptors play a crucial role in governing the immune response against most diverse viruses.

Firstly, we showed that during acute course of DENV-infection, a Th1/Tc1 profile occurs that is characterized by CCR5 expression on both CD4 and CD8 T cell populations. An interesting result is an increased Tc1-related CCR5^+^ cells among CD8 T cells in most severe patients differentiating from those without warning signs. Latter findings are contrary to most of previous studies that described Dengue Hemorrhagic Fever characterized by a Th2-skewed immune response while a Th1-skewed response was present in Dengue Fever when circulating factors are measured in sera. [44]. Contrastingly a previous report from Kadhiravan and colleagues [45] observed higher intracellular CCL3/MIP-1α positive CD8 T cells in Dengue Hemorrhagic Fever patients, a chemokine known to bind to CCR5 and to skew the immune response towards a Th1 polarity [46]. Thus, cytokine levels in serum do not necessarily reproduce the pattern at intracellular level neither in the total T-cell response nor in Th1, Th2-related marker receptors, such as chemokine receptors.

It is believed that in vitro, CD4 Th1 polarized cells “preferentially” express CCR5, CXCR3 and CXCR6, although CD4 T cells generated in vivo show a much more complex chemokine receptor profile. Moreover, the efficacy of the specific immune response is due to T cell clones that are able of performing multiple effector functions such as production of different cytokines and chemokines, activity of costimulatory molecules, and capacity to degranulate and to express cytotoxic molecules such as perforin [47], [48], [49]. Apart from a helper activity, CD4 T cells can exert a cytotoxicity activity that has been described during persistent infections such as those by Epstein-Barr virus [50], cytomegalovirus [51] or BK polyomavirus [52]. A report using a clinical approach in a study about children suffering from dengue infection was published recently and showed that in both primary and secondary Dengue Hemorrhagic Fever, <20% of T cells produce CD107a, whereas >80% produce cytokine only. These events are reversed in primary Dengue Fever where 71% produce CD107a and 29% cytokine only in CD8+ and in secondary infection 45% T cells produce CD107a. The CD107a expression was found on CD4+ T cells in addition to CD8+ during dengue fever, an observation that fits with cytotoxicity reports from anti-dengue CD4+ T-cell lines, indicating that this cell population might play an important in vivo cytotoxic role [53]. During our study, in the acute DENV-patients, the degranulation marker CD107a, besides being expressed in a substantial fraction of CCR5+ CD8+ T lymphocytes, it is expressed as well in CCR5+ CD4+ T cells [54]. In addition, “Th1” chemokine receptor CCR5+ T cells, CCR5+CD107a+ expressing T cells predominate in the response of DENV-patients, independently of severity, and CCR5+CD107a^neg^ expressing T cells are minority. Thereafter, we intend to further characterize the cytotoxic functionality of CD4 and CD8 T lymphocytes from DENV-patients and their eventual role in controlling viral spreading.

The receptor for the IFN-γ-inducible CXC chemokines is CXCR3 and it is mainly expressed on activated CD4^+^, memory/activated CD8^+^ and NK cells [55], [56]. Similarly to CCR5, CXCR3 expression is closely linked to Th1 function [57]. The ligands for CXCR3 are CXCL9/Mig, CXCL10/IP-10 and CXCL11/I-TAC are induced by the Th1 cytokines IFN-γ and TNF-α [58], [59], [60]. The activity of IFN-γ-inducible CXC chemokines mediated via CXCR3 was firstly investigated using a model of DENV-induced neurological disease in wild-type (WT), CXCR3-or CXCL10/IP-10 deficient mice [61]. In CXCR3^−/−^ mice and more expressively in the CXCL10/IP-10^−/−^, DENV-infection presented a significantly higher mortality rates than the wild-type (WT) mice. Moreover, the brains of CXCR3^−/−^ mice showed higher viral loads and quantitatively fewer T cells, in particular fewer CD8 T cells than those of WT mice, indicating that CXCR3 plays a protective role in DENV infection. Later, Ip and Liao [62], [63] found that viral loads were higher in the brains of CXCL10^−/−^ mice than in WT mice and unexpectedly, CXCL10^−/−^ mice showed comparable numbers of total infiltrating T cells, higher numbers of CXCR3+ T cells, and higher numbers of antibody-secreting cells in the brain than WT. Additionally, CXCL10 was induced in neurons following DENV infection and inhibited DENV binding to cell surface heparan sulfate, a co-receptor for DENV entry. These results demonstrate that the enhanced susceptibility of CXCL10^−/−^ mice to DENV infection is not due to a defect in recruitment of effector lymphocytes but rather to a defect in the antiviral activity that promotes viral clearance. We found in this report a higher frequency of CXCR3+ among CD4 T cells in the acute DENV-infection, while a similar proportion of the CXCR3+ among CD8 T cells was detected in DENV-infected patients as compared to controls. No correlation was found between CXCR3-expressing cells among T lymphocytes with frequency of total T cells in the blood, suggesting that CXCR3 might play a minor part in T-cell trafficking toward peripheral tissues. Moreover, the average frequency of cells displaying the degranulation marker CD107a on the peripheral CXCR3 among CD4 and CD8 T cells were significantly higher in acute DENV-infection than in convalescent patients or controls. In accordance with DENV infection in murine models, we presume that CXCR3 would play a more immunoprotective role rather than immunopathological in DENV-infection.

Dejnirattisai and colls. (2008) observed that activated T cells from dengue patients were able to overcome inhibited maturation from DENV-infected dendritic cells (DC), suggesting that activated T cell-derived soluble factors or direct cell-to-cell contact was required for DC maturation. In this system, DENV-specific T cells co-cultured with DCs produced IFN-γ, implying in enhanced IFN-γ-dependent responses such as CXCL9, CXCL10, and CXCL11 production. These factors interact with CXCR3 displayed on memory and activated T cells, especially IFN-γ-producing T cells, and promote cell chemotaxis to infection site. Interaction of these cells with the immature DENV-infected DC will now lead to a different outcome.

In this same line, a study of Rossi et al., 2011 showed that CCR5 antagonist might have a potential role in the down-regulation of HIV-associated chronic inflammation by blocking the recirculation and trafficking of monocytes, macrophages and monocyte-derived dendritic cells. In dengue, the CXCR3, and as well as CCR5, could both be important in cell traffic once these receptors are found on memory and activated T cells and promote their chemotaxis, besides facilitating the interaction of T cells with DENV-infected DC or monocytes, ultimately influencing the disease severity.

CCR4 is selectively expressed on the majority of peripheral memory Th2 cells and antigen presenting cells that produce CCL17/TARC and CCL22/MDC [24]. Its expression is increased in most skin-homing Th2 cells that express cutaneous lymphocyte-associated antigen (CLA) [64], [65]. Recently, Guabiraba and colls. [66] observed that activation of CCR1, CCR2 and CCR4 in a dengue murine model has discrete roles in the pathogenesis of the infection and that the chemokine storm that follows severe primary dengue infection is associated mostly to development of disease rather than protection. Our results indicated that contrasting to CXCR3, CCR4+ among T cells were increased only in CD8 T cells during DENV infection but no significant change in the expression of these markers was detected for CD4 T lymphocyte subsets. Then, we have investigated the phenotype and function of human T cells expressing CCR4 emphasizing CD8 T cells. The differentiation of T cells is accompanied by the acquisition of distinct chemokine receptor expression and migratory abilities by T cells. We observed earlier Integrins present at the lymphocyte surface such as the CD29 (β_1_) integrin that was up-regulated on CD4 and CD8 T cells during dengue infection [67]. Naïve CD8 T cells express CD127, but during acute viral and bacterial infection, as activated CD8 T cells expand most cells down-regulate CD127 [68]. The small subset of effector cells that expresses CD127 (referred to as IL-7Rα^high^) preferentially survives and matures into memory CD8 T cells that express increased amounts of CD27, Bcl-2, and IL-2 and undergo IL-15-driven homeostatic turnover. In contrast, most IL-7Rα^low^ effector CD8 T cells disappear over this time [69]. Thus, following acute dengue and until convalescence, CD127 expression did not change in CD4 T cells, but this molecule was down-regulated in CD8 T cells that may be considered here as effector CD8 T cells. We also showed that CCR4 was expressed on a percentage of the effector CD8 T cell population that had the ability to migrate since CD29 and CD44 were up-regulated, whereas CCR4 bearing CD4 T cells indicates that such cells belong to a less differentiated subset (no change in CD29 and CD44). Concerning the CCR4+CD8+ T cells, Kondo and Takiguchi [25] showed that these memory T cells had the ability to produce multiple cytokines but not to express perforin, granzyme A nor B. Although we have not studied the profile of intracellular cytokines from these cellular subsets, the poor CD38 or CD107a labeling indicates the low cytotoxicity of these cells (data not shown). In DENV-murine models, authors discussed the differential role of CD8 T cells and CCR4-expressing cells on the control of viral entry and replication or tissue and systemic damage. In fact, CD8^+^ T cell depletion resulted in a higher viral load in the serum, spleen, and brain. On the other hand, depletion of CCR4 did not play a major role in the control of viral entry and replication, but decreased the tissue and systemic damage. In human models, our results found that CCR4+ T cells, although the cells have phenotype effector/memory, exert their effects on the control of viruses in blood, with no injury to tissues or organs.

On the other hand, CCR4 depletion did not play a major role in the controlling of viral entry and replication, but decreased the tissue and systemic damage. In human models, our results showed that CCR4+ T cells, despite having effector/memory phenotype, they may take part in virus clearance in blood, without causing injuries to tissues or organs.

Here, we determined that circulating CCL5/RANTES levels were significantly lower in acute DENV-infected patients than in healthy controls. In the study by Chau et al in 2008, plasma RANTES levels were significantly lower in infants with acute dengue than in infants with other febrile infections and RANTES concentrations were negatively correlated with aspartate aminotransferase levels. [33].The authors might be possibly suggesting a relationship between hepatic dysfunction and these chemokines that participate in the recruitment of lymphocytes to sites of infection. In our study, we found no correlation between CCL5/RANTES and transaminases, but we observed that there is a decrease in RANTES plasma levels in patients during acute phase that is associated with the lower platelet numbers or with thrombocytopenia that is an important indicator of disease severity. The literature published on the RANTES involvement in the immunopathogenesis of human dengue fever is very little, hindering a further discussion of their role in the disease. In previous studies CCL2/MCP-1 was related with hypotension and thrombocytopenia, contrasting to CCL4/MIP-1β that was associated with a good prognosis [70]. In the current studied patients, both Th2 or Tc2-related CCL2/MCP-1 and Th1 or Tc1-related CCL4/MIP-1β discriminated well the mild DF from the severe patients, in which both markers showed decreasing levels with disease severity increase.

Interestingly, our results show a significant relationship between the CCR4 frequencies among T cells with increasing circulating CD8 T cells. In contrast, the increase of CCR5-expressing T cells or their most specific ligand cognate CCL4/MIP-1β is associated with the decrease of CD4+ T cell frequency in the blood. Therefore, it is possible that the CCR5 inflammatory chemokine receptor expression enhances the CD4+ T cell recruitment into lymph node recirculation or in inflamed sites. In support to these events there is evidence that evaluation of liver tissue specimens from four dengue fatal cases indicates the increased CCL5/RANTES expression in resident cells during dengue infection.

Briefly, our results suggest that in acute DENV-infection the reference profile responses are Th1 associated with a balance in Tc1/Tc2 responses, whereas in convalescence there is a Th1 dominance. The Th1-related CCR5- or CXCR3-expressing CD4 T cells are known in the literature to be associated with the effector or memory stage and more interestingly, in acute DENV-infection these cell subsets increased CD107a expression, characteristically of the potential ability to perform activities such as cytotoxicity. In other hand, both Tc1-related CCR5 and Tc2-related CCR4 among CD8 T cells showed an increase in CD107a expression and in effector/migratory markers as CD29, CD44^HIGH^ and CD127^LOW^ respectively, indicating their potential function during acute DENV-infection. Furthermore, the CCR5+ cells among T lymphocytes would enhance the CD4+ T cell recruitment to inflamed sites such as liver, as it was evidenciated by the increased hepatic expression of their cognate ligand CCL5/RANTES in fatal cases.

Our data strongly indicate that during natural infection by DENV, chemokine/chemokine receptors were intensely modulated. The impact of this modulation points to contradictory effects. An increased frequency of chemokine receptors among peripheral T lymphocytes is associated with effector cell capacity, as an antiviral immune response. In addition, differential amounts of chemoattractant chemokines in the circulation or in tissues suggest that the peripheral immune cells migrate, probably in order to control viral infection in inflammation/inflamed sites. On the other hand, the activation of immune cells during dengue infections also contributes to induce pathogenesis as well as described in the literature. Therefore, the real impact of our preliminary descriptive data on the balance between immunoprotection versus immunopathogenesis is under evaluation by our team and deserves to be further investigated by others as well.
